# Psilocybin increases emotional empathy in patients with major depression

**DOI:** 10.1038/s41380-024-02875-0

**Published:** 2024-12-18

**Authors:** J. Jungwirth, R. von Rotz, I. Dziobek, F. X. Vollenweider, K. H. Preller

**Affiliations:** 1 Department of Adult Psychiatry and Psychotherapy, Psychiatric University Clinic Zurich and University of Zurich, Zürich, Switzerland; 2 Clinical Psychology of Social Interaction, Department for Psychology, Humboldt-Universität zu Berlin, Berlin, Germany

**Keywords:** Depression, Psychology

## Abstract

Empathy plays a crucial role in interpersonal relationships and mental health. It is decreased in a variety of psychiatric disorders including major depression. Psilocybin, a promising candidate for treating depression, has been shown to acutely increase emotional empathy in healthy volunteers. However, no study has investigated this effect and its relevance for symptom improvement in a clinical population. This study examines the enduring effects of psilocybin-assisted therapy on empathy in depressed patients using a randomized, placebo-controlled design. Fifty-one depressed patients were randomly assigned to receive a single dose of psilocybin (0215 mg/kg body weight) or a placebo embedded in a 4-week psychological support intervention. Empathy was measured using the Multifaceted Empathy Test at baseline and 2 days, 1 week, and 2 weeks after substance administration. Changes in empathy were compared between treatment conditions. Patients who received psilocybin showed significant improvements in explicit emotional empathy driven by an increase in empathy towards positive stimuli compared to the placebo group for at least two weeks. This study highlights the potential of psychedelics to enhance social cognition in individuals living with depression and contributes to a better understanding of the psychological mechanisms of action of psychedelics. Further studies are necessary to investigate the interaction between social cognition and clinical efficacy.

The trial is registered on clinicaltrials.gov (Identifier: NCT03715127) and KOFAM (Identifier: SNCTP000003139).

## Introduction

Empathy is a key aspect of social cognition and considered critical for healthy social interaction [1, 2]. Social cognition is crucial for successfully managing every-day life (e.g. work environment, education, relationships, friendships) but is also a critical component of therapeutic processes [1]. Difficulties in this domain can play a significant role in developing and maintaining psychiatric disorders [1, 3]. Clinical research demonstrates that empathy is impaired in depression, alcohol use disorder and other psychiatric disorders [4–17]. Psychiatric treatments rarely address difficulties in social cognition and therefore improvements in this domain remain a large unmet need [1].

Empathy is the ability to vicariously experience and understand the feelings of others. Two main aspects of empathy can be distinguished: *emotional* and *cognitive empathy* [2]. *Emotional empathy* defines the ability to *feel* what another person is feeling, i.e., an observer has an emotional response to the emotional state of another person [18, 19]. Cognitive empathy, also referred to as “theory of mind”, represents the ability to *know* or label what another person is feeling, i.e., taking the perspective of an observed person and deducing their mental state [16, 20, 21].

Psilocybin (4-phosphoryloxy-N,N-dimethyltryptamine) is a classic psychedelic that induces dose-dependently an altered state of consciousness. The subjective effects are mediated primarily through agonism at 5-HT-2A and 5-HT-1A receptors [22]. Within the so-called renaissance of psychedelic research and the urgent search for new treatment options for depressive and other psychiatric disorders, psilocybin represents one of the most investigated candidates [23]. It showed promising results in the treatment of depression [24–28], end-of-life anxiety [29, 30] and alcoholism [31]. In the present study a remission-rate of 54% was observed in the psilocybin group [28]. However, the mechanisms of action and its relationship to clinical outcomes are still poorly understood.

Previous research demonstrated that psilocybin increases emotional empathy during its acute effects in healthy volunteers [32]. Furthermore, a recent study showed an increase in emotional empathy the morning after psilocybin intake in an unblinded retreat setting in healthy volunteers. Seven days later, increases in emotional empathy remained significant for negative emotions [33]. No changes in cognitive empathy were detected in these studies.

Given the importance of social cognition in psychiatric disorders, psilocybin-induced modulation of empathy represents a candidate psychological mechanism that may contribute to the efficacy of psilocybin-assisted therapy [34]. However, no study to date has investigated the lasting effects of psilocybin on empathy in a placebo-controlled design or a clinical population.

Therefore, the present study investigates the effect of a single dose of psilocybin vs. placebo on empathy in depressed patients at 2 days, 8 days, and 14 days post-treatment. We tested the following hypothesis: Psilocybin compared to placebo increases emotional empathy for up to two weeks after administration. To further characterize the relationship between empathy and clinical outcomes, we hypothesized that improvements in empathy are associated with improvements in depressive symptoms independent of treatment condition.

## Methods

### Study design

This randomized, double-blind, placebo-controlled, parallel-groups trial involved 7 in-person visits over 4 weeks for each participant. The data were collected as part of a clinical trial testing the efficacy of psilocybin-assisted therapy for major depression [28]. The pharmacological intervention consisted of a single oral administration of either psilocybin (0.215 mg/kg body weight) or placebo (mannitol). Participants received 2 psychological preparation sessions one day and four days before substance administration to prepare them for the treatment, ensure participant safety, set a therapeutic goal, and foster a trusting therapeutic relationship. Two, eight, and fourteen days after psilocybin administration, 3 integration sessions were conducted to help process difficult emotions, to develop a meaningful narrative of the experience and support for making adequate behavioural adjustments in daily life. All psychological support sessions were provided by the same study therapist and lasted around 60 min. For details regarding psychological support, see supplementary information and von Rotz et al. [28]. Participants in both intervention arms completed identical procedures during each visit. The trial was conducted in German, corresponding validated versions of questionnaires and interviews were used. Approval was granted by the Cantonal Ethics Committee Zurich, Switzerland, the Swiss Agency for Therapeutic Products (Swissmedic), and the Federal Office of Public Health (FOPH). The study is registered on clinicaltrials.gov (Identifier: NCT03715127) and KOFAM (Identifier: SNCTP000003139) and was conducted in accordance with the Revised Declaration of Helsinki (2000) and the International Council for Harmonisation Good Clinical Practice (GCP) guidelines. Synthetic, pharmaceutical-grade psilocybin was obtained from Compass Pathways Ltd., United Kingdom. All participants and study staff, with the exception of the resident pharmacist, were blinded to the treatment assignments until the database was closed. Additional information on the study procedure is provided in the supplement.

### Sample characteristics

Eligible for participation were women and men between the ages of 18 and 60 years meeting DSM-IV criteria of a current episode of mild or moderate depression. Detailed inclusion and exclusion criteria as well as recruitment methods and symptom severity are outlined in the supplement.

After referral or outreach, participants were pre-screened via telephone by a trained member of the study team. A structured interview was conducted on psychiatric history, sociodemographics, and somatic health. General information about the study was provided. Potential participants were then invited for a medical and psychiatric screening on-site. Written informed consent was obtained from all participants prior to medical screening. Any psychiatric medication was washed out at least 2 weeks or five half-lives before administration day and participants consented to pause any external psychotherapy for the duration of the study to minimize confounding factors (for details see von Rotz et al. [28]). The Mini International Neuropsychiatric Interview (M.I.N.I.) was conducted to confirm the current depressive episode within a major depressive disorder and to diagnose and rule out other psychiatric disorders. Symptom severity of the depressive episode was measured using the Montgomery-Åsberg Depression Rating Scale (MADRS) and Beck’s Depression Inventory (BDI). For inclusion, potential participants required a MADRS score between 10 and 40 (out of 60) points.

### Measures

#### Multifaceted Empathy Test (MET)

The MET is a computer-assisted test showing 40 photos of people embedded in various social or non-social contexts displaying different complex emotions. Twenty photos are categorized as negative stimuli (depicting sadness, anger, shock etc.) and 20 photos as positive stimuli (depicting joy, feeling of security, calm etc.). Every photo is shown 3 times with 3 different questions to assess the different components of empathy. Cognitive empathy is tested asking the participant which emotion the person in the photo is feeling. One correct answer needs to be chosen out of 4 options. Implicit emotional empathy is assessed by asking „How aroused does this picture make you feel?”. A 9-point Likert-scale is used to answer this question (1 = very calm; 9 = very aroused). Explicit emotional empathy is assessed by the question “How strongly do you feel for this person?”. Another 9-point Likert-scale is used to answer this question (1 = not at all; 9 = very strongly). Thus explicit emotional empathy can be regarded as an equivalent to empathic concern. The MET is considered a valid, reliable, and naturalistic method able to reduce major biases (e.g. social desirability) [11, 14, 20, 35]. Patients completed the MET four times: At -1d (one day prior to the drug intervention, baseline), and +2 d, +8 d and +14 d after psilocybin administration.

#### MADRS

The Montgomery-Åsberg Depression Rating Scale (MADRS) is a widely used clinician-rated instrument for the assessment of the severity of depression. The scale consists of 10 items, each rated on a scale of 0–6, with higher scores indicating greater severity of the symptom. The items assess various aspects of depression, including mood, cognitive symptoms, sleep and others. The MADRS has been found to have good reliability and validity in several studies and is used in both clinical and research settings [36]. It was assessed at every study visit by the same attending study therapist.

#### BDI

The Beck Depression Inventory (BDI) is a common self-report questionnaire for the assessment of the severity of depression. The scale consists of 21 items, each rated on a scale of 0-3, with higher scores indicating greater severity of depression. Similar to the MADRS the items assess various aspects of depression. With proven reliability and validity, BDI is applied in clinical and research settings [37]. It was assessed at every study visit.

### Statistical analysis

Data analysis of empathy scores (MET), clinical endpoints (MADRS, BDI), and secondary endpoints was performed on the intention-to-treat population (ITT), i.e., all participants who received psilocybin or placebo treatment and had at least one assessment of empathy and efficacy post-treatment. Empathy scores were not imputed. In contrast, missing efficacy scores (i.e. MADRS, BDI) were imputed by using the last observation carried forward method for the purpose of maximizing statistical power for the analysis of associations between clinical response and changes in empathy and maintaining consistency in reporting with the primary efficacy analysis reported elsewhere [28]. We conducted various sensitivity analyses on the raw data to identify potential validity issues to ensure that our key findings remained robust and consistent, with none of the assessments being excluded from the analysis.

Mixed effects analyses of variance were calculated for all MET subscales. Significant interaction terms between treatment condition (between-subject factor) and visit number (within-subject factor) lead to subsequent post-hoc testing. The Greenhouse-Geisser correction was applied where necessary to account for violations of sphericity, leading to fractional degrees of freedom in some cases. Generalized eta squared (η^2^_G_) is considered the appropriate measure to report the amount of variance explained by the model. Correction for alpha level inflation was achieved using the Benjamini-Hochberg method, adjusting *p*-values from ANOVA interaction terms for each subscale and valence level. In addition, Student’s *t*-tests were used to determine post-hoc significance in differences between treatment conditions for each study visit and resulting *p*-values were adjusted for *N* = 3 comparisons (three post-intake timepoints times; positive valence in Fig. 2). Cohens’ *d* was used to estimate the magnitude of group difference effect sizes. All statistical tests used *P* < 0.05, two-tailed, to determine statistical significance. As previous publications point to valence-specific effects of psilocybin on empathy [32, 33], this analysis was repeated for negative and positive stimuli separately.

Pearson correlation analysis was conducted to test the association between changes from baseline (post-pre administration) in explicit emotional empathy and changes from baseline in depressive symptoms assessed by MADRS and BDI. Higher change scores for MET subscales indicate increased levels of empathy, while positive change scores in depression severity reflect a worsening of symptoms. Fisher’s z-Test was calculated to test for differences in the correlations for each treatment group. *P*-values from Pearson correlations and Fisher’s z-Tests were adjusted using Holm’s method to account for *N* = 6 comparisons.

Statistical analyses were performed using the rstatix package in R. Visualization of the data was done with R packages lattice, ggpubr, and ggplot2 [38–42].

## Results

A total of 1152 individuals were initially screened via mail or phone. Out of these, 68 individuals experiencing an acute depressive episode were invited for medical screening. 55 met all the necessary criteria and were therefore enrolled in the study. Before randomization, three individuals opted out due to logistical problems related to the Covid-19 pandemic, leaving 52 participants to receive the drug treatment. During +2 d one additional participant in the psilocybin group withdrew from further study participation wishing to get back on medication before completing the first MET follow-up assessment and was therefore not included into the analysed sample. A total of 51 participants was therefore included in the analysis (Supplementary Fig. 1). Two patients were lost to follow-up (one at +8 d and one at +14 d). In addition, one MET assessment at +2 d, four at +8 d and three at +14 d were excluded from analysis due to technical issues with the experiment. For details on demographic information see Table 1 and supplement.

**Table 1:** Demographic information of *N* = 51 participants.

		Placebo	Psilocybin
Sample Size		*N* = 26	*N* = 25
Gender	female	17 (65.4%)	15 (60.0%)
	male	9 (34.6%)	10 (40.0%)
Age	Mean (SD)	35.9 (9.80)	37.6 (11.1)
	Median [Min, Max]	33.5 [21.0, 57.0]	34.0 [22.0, 60.0]
Ethnicity	Arab	2 (7,7%)	0 (0,0%)
	Black Caribbean	1 (3,9%)	0 (0,0%)
	White	23 (885%)	25 (100%)
Verbal IQ (at medical screening)	Mean (SD)	113 (12.1)	115 (13.0)
	Median [Min, Max]	112 [97.0, 143]	112 [97.0, 143]
CGI severity (at medical screening)	Mean (SD)	5.54 (0.905)	5.52 (0.586)
	Median [Min, Max]	5.50 [4.00, 7.00]	5.00 [5.00, 7.00]
MADRS score (at medical screening)	Mean (SD)	23.8 (5.99)	23.9 (4.62)
	Median [Min, Max]	23.0 [13.0, 36.0]	22.5 [16.0, 35.0]
BDI score (at medical screening)	Mean (SD)	27.0 (10.3)	26.8 (6.83)
	Median [Min, Max]	25.5 [8,49]	26.0 [12,39]
Cognitive Empathy [%] (at baseline; -1d) *Positive Stimuli*	Mean (SD)	0.69 (0.09)	0.70 (0.12)
	Median [Min, Max]	0.65 [9,16]	0.75 [8,18]
Cognitive Empathy [%] (at baseline; -1d) *Negative Stimuli*	Mean (SD)	0.66 (0.11)	0.62 (0.11)
	Median [Min, Max]	0.68 [10,17]	0.60 [9,18]
Emotional Empathy (at baseline; -1d) *Positive Stimuli*	Mean (SD)	4.17 (1.56)	4.29 (1.33)
	Median [Min, Max]	4.23 [1, 6.85]	4.38 [1, 6.85]
Emotional Empathy (at baseline; -1d) *Negative Stimuli*	Mean (SD)	5.05 (1.59)	5.60 (1.27)
	Median [Min, Max]	5.25 [1.25, 8.25]	5.83 [2.65, 7.75]

Verbal IQ was assessed by the Multiple-Choice Word Test (MWT-B). Clinical Global Impressions (CGI) provides a clinician-rated impression of overall severity of clinical symptomatology. Both were assessed at medical screening (≥ -5d). Percentages may not sum up to 100% due to rounding. Cognitive and emotional empathy were computed at baseline (-1d prior to drug intake) for both positive and negative stimuli for each treatment condition respectively. Note that cognitive empathy values were transformed from absolute to percentage scores in order to improve comparability to other findings in the literature.

In comparison to previous research, our baseline empathy findings are consistent with cognitive and emotional empathy ability observed in other studies of both depressed and healthy individuals [17, 35]. Specifically, although our cognitive empathy baseline scores (mean = 0.67) were slightly lower than those reported by Guhn et al. for persistent depressive disorder (PDD) (0.78) and Major depressive disorder (MDD) (0.79) and by Foell et al. for Healthy Controls (0.71), they are in the same order of magnitude and represent comparable values. Similarly, our emotional empathy scores (meanpos = 4.2, meanneg = 5.3) closely resemble those reported for patients with MDD (meanpos = 4.3, meanneg = 5.7) and PDD (meanpos = 3.8, meanneg = 5.3), indicating a similar emotional empathy profile.

### Effects of psilocybin on emotional and cognitive empathy

**Fig. 1:**
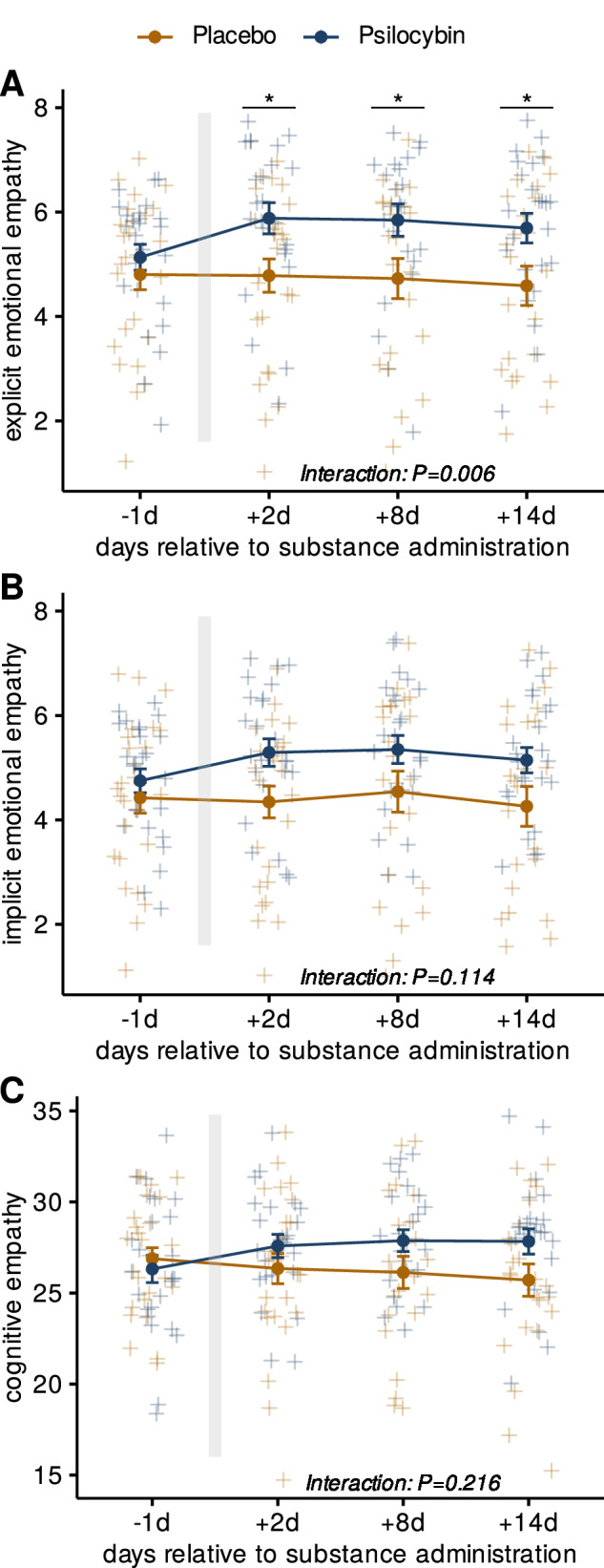
Changes in MET scores over time for psilocybin and placebo groups. **A** Explicit emotional empathy. **B** Implicit emotional empathy. **C** Cognitive empathy. All panels depict mean scores at -1d, +2 d, +8 d and +14 d relative to substance administration. The grey bar depicts the day of substance administration. Differences between treatment conditions were analyzed using mixed measures ANOVA. Post-hoc Student’s *T*-tests were conducted to compare treatment conditions and yielded significant differences for all study visits post-administration (Benjamini-Hochberg-corrected; P <  0.05) for explicit emotional empathy. Error bars represent standard errors of means (se); blue = psilocybin; yellow = placebo; *p < 0.05; *N* = 51.

Mixed effects models comparing MET scores for each empathy subscale resulted in a significant interaction between treatment condition and study visit for explicit emotional empathy F(2.43, 102) = 5.83; *P* = 0.006; η^2^_G_ = 0.01, but not for implicit emotional empathy F(2.21, 92.7) = 2.57; *P* = 0.11; η^2^_G_ = 0.005, or for cognitive empathy F(3, 126) = 2.39; *P* = 0.22, η^2^_G_ = 0.013. Subsequent post-hoc analysis of explicit emotional empathy scores revealed significant differences between treatment conditions for all study visits after substance administration (*P* < 0.05). Strongest numeric differences between treatment conditions for explicit emotional empathy were found 8 days after the intervention (1.12 points; 95% C.I. = [0.13, 2.11]; *P* = 0.027; *d* = 0.66). Results for each empathy subscale are depicted in Fig. 1.

### Increased explicit emotional empathy for positive emotions after psilocybin administration

**Fig. 2:**
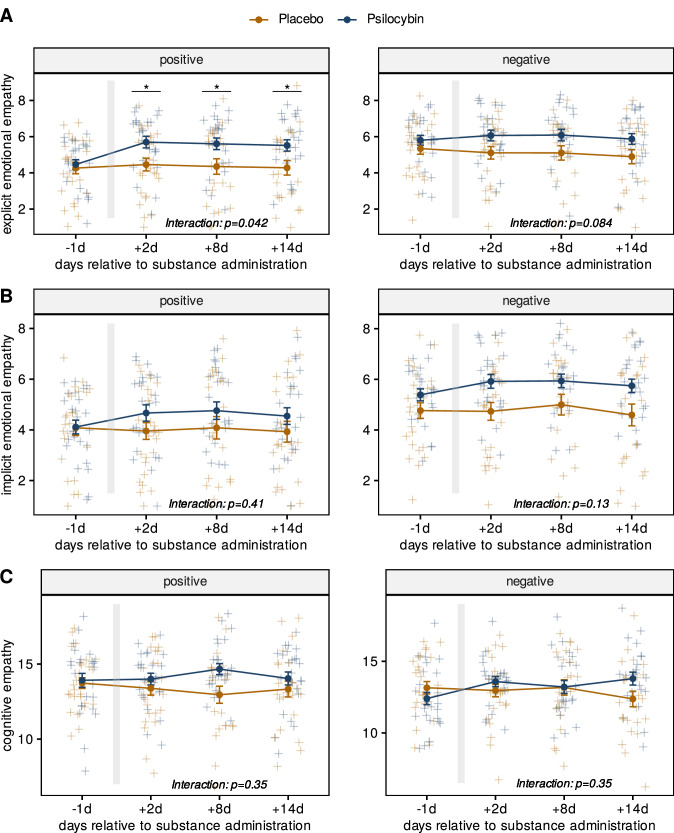
Changes in MET scores for positive and negative stimuli. **A** Explicit emotional empathy. **B** Implicit emotional empathy**. C** Cognitive empathy. All MET scores are depicted for the psilocybin and placebo groups at -1d, +2 d, +8 d and +14 d relative to substance administration. The right panels show MET scores in response to negative stimuli, the left panels show MET scores in response to positive stimuli. The grey bar depicts the day of substance administration. Differences between treatment conditions at each time point were calculated using mixed measures ANOVA. Differences between treatment conditions for individual visits were calculated using Student’s *T*-test with Benjamini-Hochberg-adjusted *p*-values at *P* < 0.05. Only explicit emotional empathy in response to positive stimuli showed a significant interaction allowing for post-hoc testing to compare treatment conditions at each study visit. Error bars represent standard errors of means (se). blue = psilocybin; yellow = placebo, *p < 0.05, *N* = 51.

Separate mixed effects models for positive and negative stimuli comparing MET subscales between treatment conditions are shown in Fig. 2. Significant treatment group * study visit interactions were obtained for explicit emotional empathy scores for positive stimuli [F(2.55, 107) = 4.62; *P* = 0.042; η^2^_G_ = 0.012], but not for negative stimuli [F(2.15, 90.2) = 3.62; *P* = 0 .084; η^2^_G_ = 0.006].

In addition, an exploratory analysis comparing baseline empathy scores for negative versus positive stimuli revealed that patients displayed significantly higher baseline scores for explicit empathy in response to negative stimuli (1.20 points; 95% C.I. = [0.63; 1.78], *P* < 0.001, *d* = 0.82) as well as implicit empathy in response to negative stimuli (0.97 points; 95% C.I. = [0.41; 1.53], *P* < 0.001, *d* = 0.68).

For positive stimuli significant differences were found for all three study visits post-administration, with only marginally varying estimates between +2 d (1.24 points; 95% C.I. = [0.27; 2.32]; *P* = 0.042; *d* = 0.73) and +14 d (1.25 points; 95% C.I. = [0.20; 2.26]; *P* = 0.042; *d* = 0.70). No further significant interactions were obtained (all *p* > 0.05).

### Association between changes in explicit emotional empathy and depressive symptoms

**Fig. 3:**
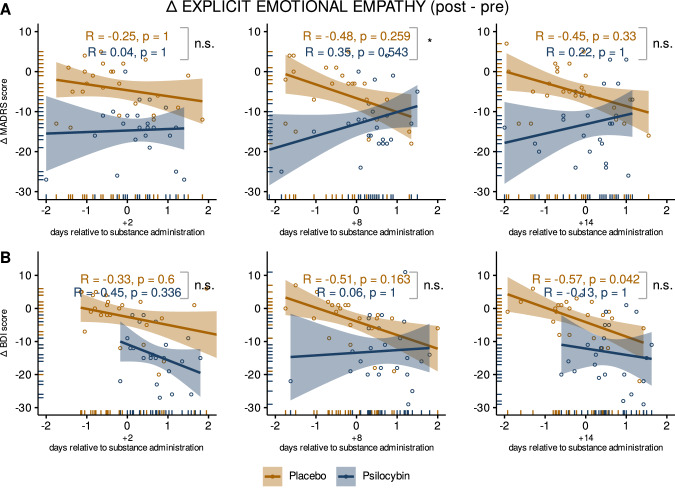
Correlations between MET and changes in depressive symptoms. Associations between change from baseline for MADRS (**A**) and BDI (**B**) and changes from baseline for explicit emotional empathy scores for both treatment conditions for the three timepoints after drug administration (left = +2 d, center = +8 d, right = +14 d). The X-axis shows the change in explicit emotional empathy scores from baseline (post-pre administration). Y-axis shows the change in depressive symptoms from baseline as measured by MADRS and BDI (post-pre administration). Blue represents the psilocybin condition (*N* = 25), and yellow the placebo condition (*N* = 26). Correlation coefficients (R) were calculated using the Pearson correlation method, *p*-values were adjusted for *N* = 12 comparisons using the benjamini-hochberg’s method and are displayed per treatment condition. Regression lines include 95% confidence intervals. Fisher’s Z test was used to identify statistical differences between the correlations in each treatment condition (square parentheses) and *p*-values were adjusted for *N* = 6 comparisons. Dots represent individual measurements. *P < 0.05; **P < 0.01; ***P < 0.001.

When testing the relationship between changes in depression scores and changes in explicit emotional empathy scores, significant associations were observed in the placebo group at +8 d on the MADRS and at +14 d on BDI (Fig. 3; both *P* < 0.05, corrected). No significant associations were observed in the psilocybin group (all *P* > 0.05, corrected). After correction for multiple comparisons all *p*-values from Fisher’s z-Test, testing for differences in correlations between groups, were found to be *p* > 0.05.

## Discussion

The present study investigates the effects of psilocybin on empathy in depressed patients using a randomized, placebo-controlled design. Our results show that psilocybin has significant positive effects on explicit emotional empathy in depressed patients. These effects were sustained 2 weeks after psilocybin administration, highlighting a potential long-lasting impact of psilocybin on social cognition in depressed patients. This effect was significant for positive stimuli across all time-points, but not for negative stimuli. At baseline participants showed significantly stronger empathy towards negative compared to positive stimuli, which underscores the well-documented tendency for depressed patients to exhibit heightened sensitivity to negative emotional stimuli [17, 43]. The observation that individuals with depression are more likely to recognize and resonate with negative emotions is called negativity bias [43]. Considering that psilocybin appears to enhance emotional empathy while simultaneously reducing depressive symptoms, the absence of a significant effect on empathy toward negative stimuli may align with theoretical expectations. Previous studies with healthy participants respectively showed acute [32, 44] and sub-acute [33] increases in emotional empathy after psilocybin and LSD administration towards both - positive and negative stimuli. A preliminary open-label study with healthy participants receiving ayahuasca (containing the psychedelic N,N-Dimethyltryptamine) showed increased implicit emotional empathy towards positive stimuli, which also correlated with improvement on the Satisfaction with Life Scale at 7 days after drug administration [45]. This positive correlation between increased emotional empathy towards positive stimuli and increased Satisfaction with Life was also reported in healthy participants receiving psilocybin [33].

In contrast to these findings, no significant correlations between increased empathy and improvement in depressive symptoms were found in the present study. Hence, although this improvement in emotional empathy may add to the overall psychosocial improvement in depression, our findings do not allow the conclusion that improvement in empathy is a key psychological mechanism of psilocybin which drives the reduction of depressive psychopathology. Notably, however, a significant association between changes in empathy and changes in depression was found in the placebo group, indicating a complex and likely bidirectional interrelation between empathy and depression. As depressive symptoms remit, patients may become more responsive to positive emotions, which could drive the observed increase in empathy scores. Conversely, an enhanced ability to detect and empathize with positive emotions may itself contribute to the reduction of depressive symptoms. Given this complexity, future studies should consider incorporating neutral stimuli as a control and emotional morphing paradigms to help disentangle the specific effects of psilocybin treatment on emotional valence processing from those related to general mood improvements.

On a neurobiological level the increase in emotional empathy towards positive stimuli could potentially be explained by psilocybin-induced modulations of limbic structures in combination with a decoupling of functional connectivity between the medial prefrontal and the posterior cingulate cortices - two major areas of the default mode network, implicated in self-other distinction, self-related cognition and inward-outward-directed mentalizing [46]. Enhanced empathy towards positive emotions may then promote better communication and understanding between patients and their therapists by reinforcing communication itself, potentially leading to improved therapeutic alliance - a main predictor for effective psychotherapy [47–50]. Empathy improvements could also lead to more profound patient self-awareness and emotional insight, providing possible leverage for cognitive restructuring processes and behavioural modifications. Moreover, increased empathy may specifically benefit group therapy settings, where the ability to connect with and understand the experiences of others can enhance the group cohesion and dynamic. The specific increase of emotional responsiveness to positive experiences with others could help shift patients’ focus away from depressive patterns of perception within a group therapy and therefore could facilitate a broader openness to beneficial experiences. Group therapies with psilocybin could represent a resource-efficient treatment modality [51, 52]. Additionally, increased empathy towards positive stimuli might lead to improved social functioning via positive reinforcement, strengthening social support networks and fostering interpersonal relationships, which are crucial for maintaining mental health [1, 53]. By enhancing empathy, psilocybin-assisted therapy may address an important aspect of social cognition that is often impaired in various psychiatric disorders [4–17]. Therefore, implications for the treatment of other indications should be examined in further research.

We did not observe a significant change in implicit emotional empathy and cognitive empathy. The latter is consistent with previous research in healthy populations, where cognitive empathy was decreased or did not change in an acute or sub-acute time frame [32, 33]. One possible explanation for the lack of change in cognitive empathy could be that psilocybin primarily affects emotional processing and emotional regulation, rather than the cognitive aspects of empathy [1, 54].

Although the results of this study are promising, several limitations should be noted. First, the current data represent secondary outcomes of the trial, which necessitates cautious interpretation due to its exploratory nature. The sample size was relatively small and the generalizability of the findings to real-world settings is unclear. Additionally, empathy was measured after one psychological preparation session and only over a period of 2 weeks, warranting further research on the initial value of empathy and the persistence of these effects. Moreover, the study focused on patients with mild to moderate depression, limiting the generalizability of the results to more severe symptoms or other psychiatric populations. The clinical outcome MADRS was not assessed by an independent rater, introducing potential bias to the exploratory correlation with empathy. We did not assess the expectancy and the success of the blinding during the study. Expectancy of patients in combination with an intervention that is difficult to blind can substantially alter outcomes by facilitating placebo and nocebo effects in both groups.

In conclusion, our findings provide evidence for the lasting effects of psilocybin on empathy in depressed patients, with significant increases in explicit emotional empathy observed up to 14 days after treatment. Given that conventional antidepressants have been observed to reduce empathy [55, 56], psilocybin could be a promising candidate for enhancing social cognition and strengthening therapeutic alliance. These results have important implications for the development and application of psychedelic-assisted therapy in the treatment of depression and other psychiatric disorders characterized by impaired empathy. Further research is needed to elucidate the underlying neurobiological mechanisms involved in these effects and to explore the potential of pro-empathic pathways in novel treatment strategies.

## Supplementary information

Supplementary Information

## Data Availability

Primary endpoints and demographic information collected for the study, including de-identified individual participant data and a data dictionary defining each field in the set, will be made available immediately after publication with no end date to researchers who provide a methodologically sound proposal. Proposals should be directed to the corresponding author.
