# Supplementary material for: Psilocybin increases emotional empathy in patients with major depression
Source: Mol Psychiatry. 2024 Dec 18;30(6):2665–72. doi: 10.1038/s41380-024-02875-0 (PMC12092279; doi:10.1038/s41380-024-02875-0)
Supplement: Supplementary file 1 — Supplementary Information [file 41380_2024_2875_MOESM1_ESM.docx]

**Supplementary Information**:

**Recruitment and Demographic Information:**

Participants were recruited using online advertisements on the website of the Psychiatric University Hospital Zurich, advertisements in local and national newspapers and distributed flyers in several private practitioners' practices or ambulatory clinics. Furthermore, general practitioners and psychiatrists were contacted with information about the trial. At the initial medical screening session (≥ -5 days prior to substance administration), participants provided a comprehensive range of demographic data, which is systematically presented in Table 1. This encompassed details regarding age, gender, ethnicity, levels of verbal IQ, suicidality, the scores of depressive symptoms and clinical impression and response and remission rates on the MADRS. Additionally, information on any medication taken prior to entering the trial, as well as past interactions with psychedelic substances, was collected and reported in von Rotz et al. 2023. The Mehrfachwahl-Wortschatz-Intelligenztest (MWT-B), a prevalent tool for assessing verbal intelligence, was employed to calculate individual verbal IQ scores, based on the accurate identification of German words, with a maximum achievable score of 37 points. These scores were then correlated with standard norm tables to estimate verbal intelligence, where higher scores denote superior verbal IQ.

**Study Procedures:**

To ensure consistency across all study visits and to help build a trusting therapeutic relationship, one specially trained therapist was assigned to handle all sessions for a particular participant. A research assistant was responsible for administering any questionnaires related to the study. In addition to the results reported in this manuscript, participants also took part in three one-hour sessions of functional magnetic resonance imaging, underwent four neuropsychological assessments, and provided three blood samples throughout the study. Additionally, participants completed clinical and psychological follow-up assessments, the results of which will be published at a future date.

**Inclusion Criteria:**

- Capable of giving informed consent
- Informed consent as documented by signature
- Male and female in- and outpatients 18 years to 60 years of age
- Right-handedness
- DSM-IV-diagnosis of mild or moderate major depressive episode without psychotic features (based on clinical assessment and confirmed by the SCID Interview)
- Score of ≥ 10 and ≤40 on the Montgomery-Asberg Depression Rating Scale (MADRS) at both screening and baseline visits.
- Drug free from any psychotropic medication for at least two weeks (or five weeks for fluoxetine) before enrolling in the study.
- Judged clinically not to be a serious suicide risk.
- Good physical health with no unstable medical conditions, as determined by medical history, physical examination, routine blood labs, electrocardiogram, urine analysis, and urine toxicology.
- Normal level of language comprehension and German or Swiss-German as first language
- Willing to refrain from drinking alcohol the day before testing days, from drinking alcohol and caffeinated drinks during the testing days and from consuming psychoactive substances 2 weeks before enrolling in the study and for the remainder of the study.
- Women of childbearing potential must be using an effective, established method of contraception for the entire study duration, such as oral, injectable, or implantable contraceptives, or intrauterine contraceptive devices. Note: female participants who are surgically sterilized / hysterectomized or post-menopausal for longer than 2 years are not considered as being of childbearing potential.
- Have a family member or friend who can pick them up and stay with them overnight after the psilocybin administration sessions (driving is forbidden at drug treatment days)

**Exclusion Criteria:**

- Lifetime history of bipolar disorder (I, II, not otherwise specified)
- Lifetime history of schizophrenia, schizoaffective disorder, or psychosis not otherwise specified.
- History of DSM-IV drug or alcohol dependence or abuse (except for caffeine or nicotine) within three months prior to enrollment.
- Comorbid Axis I anxiety disorder diagnoses will be permitted if they do not require current treatment.
- Family history of schizophrenia or schizoaffective disorder, or bipolar disorder type 1 (first- or second-degree relatives)
- Lifetime history of hallucinogen use on more than 10 occasions.
- Getting psychotherapeutic or psychological treatment from third parties during the study is forbidden.
- Abnormal electrocardiogram
- Any unstable illness as determined by history or laboratory tests.
- BMI <17 or >35
- Uncorrected hypo- or hyperthyroidism
- Women who are pregnant or breastfeeding or have the intention to become pregnant during the course of the study.
- Contraindications to magnetic resonance imaging (MRI safety form)
- During the study, new use, or dose changes of already existing concomitant medication without prior informing the investigators is forbidden.
- Allergy, hypersensitivity, or other adverse reaction to previous use of psilocybin or other hallucinogens
- High risk of adverse emotional or behavioral reaction based on investigator's clinical evaluation (e.g., evidence of serious personality disorder, antisocial behavior, serious current stressors, lack of meaningful social support)
- Participation in another study with the investigational drug within the 30 days preceding and during the present study.

**CONSORT flow:**


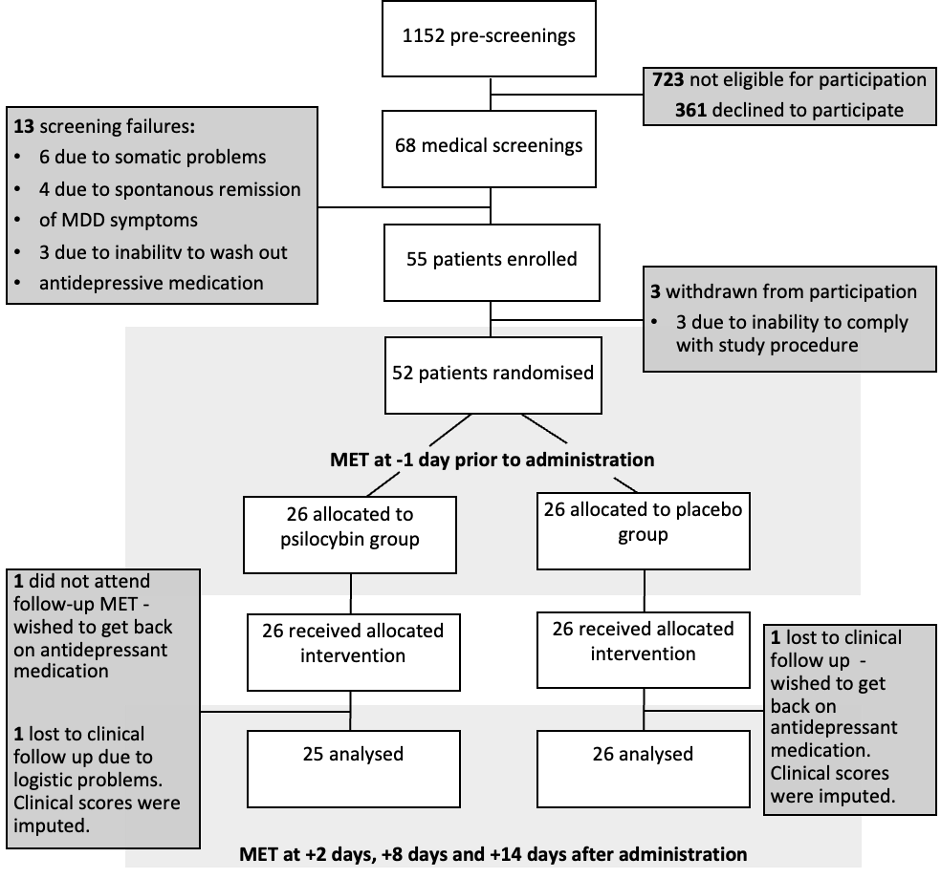


**Supplementary Figure 1** CONSORT flow diagram and study design. After enrolment, all participants underwent one Multifaceted Empathy Test (MET) and clinical measurements at -1 day prior to administration before being randomized to either the placebo or the psilocybin condition (baseline). Drug administration took place on the administration day at 9 a.m. and participants were discharged between 3 p.m. and 5 p.m. on the same day after acute drug effects had completely worn off. Follow-up METs and clinical follow-up were conducted 2 days, 8 days, and 14 days after administration. The last observation carried forward method was used to impute missing clinical efficacy values from two patients lost to clinical follow-up. Missing empathy values were not imputed.

**MADRS clinical severity:**

**Supplementary Figure 2** Number of occurrences of different MADRS severity categories (Normal, Mild, Moderate, Severe) for the two timepoints (medical screening and primary efficacy assessment [+14d]) for both treatment groups. Severity categories were defined based on MADRS scores: Normal (0–9), Mild (10–19), Moderate (20–34), and Severe (≥35). Bars represent the frequency of each severity category at the specified time points, with data grouped by treatment (Placebo, Psilocybin).
